# Supplementary material for: Stochastic Lanczos estimation of genomic variance components for linear mixed-effects models
Source: BMC Bioinformatics. 2019 Jul 30;20:411. doi: 10.1186/s12859-019-2978-z (PMC6668092; doi:10.1186/s12859-019-2978-z)
Supplement: Supplementary file 1 — Proof of result used to efficiently compute the quadratic form (7). (PDF 125 kb) [file 12859_2019_2978_MOESM1_ESM.pdf]

Let A^+ denote any generalized inverse and let $A^\dagger$ denote the Moore-Penrose pseudoinverse.

Fact 1 (Seber [2008], page 457)**.** *Let $S \in \mathbb{R}^{n\times n}$ be an orthogonal projection operator. Then for any $B \in \mathbb{R}^{m\times n}$,*

$$S(BS)^\dagger = (BS)^\dagger$$

and

$$(SB)^\dagger S = (SB)^\dagger$$

Fact 2 (Lewis and Newman [1968], Theorem 6)**.** *Let $A \in \mathbb{R}^{n\times n}$ be such that $A = A^T \succeq 0$, $C \in \mathbb{R}^{n\times c}$, and define $\tilde{A} = A + C^TC$. Let $A^+ \succeq 0$ be a generalized inverse of A. Then*

$$\tilde{A}^+ = A^+ - A^+C^T(I + CA^+C^T)^{-1}CA^+$$

is a generalized inverse of $\tilde{A}$ if and only if $\ker A \subseteq \ker C$. Further, if this is the case, we have

$$\begin{aligned}
\tilde{A}^+\tilde{A} &= A^+A,\\
\tilde{A}\tilde{A}^+ &= AA^+,\\
\tilde{A}^+ &\succeq 0,\\
\textit{and } \tilde{A}^+ = \tilde{A}^\dagger &\iff A^+ = A^\dagger.
\end{aligned}$$

Lemma 1. *Write the full QR decomposition of $X \in \mathbb{R}^{n\times c}$ as $X = QR = [Q_X|Q_{X^\perp}]R$ such that $Q_X \in \mathbb{R}^{n\times(n-c)}$, $Q_{X^\perp} \in \mathbb{R}^{n\times c}$ and define $S = I_n - Q_XQ_X^T$. Then,*

$$y^TS(S(H+\sigma I)S)^\dagger Sy = y^TS(SHS+\sigma I)^{-1}Sy$$

Proof. Applying the first fact,

$$y^TS(S(H+\sigma I)S)^\dagger Sy = y^T(S(H+\sigma I)S)^\dagger y.$$

Rewrite the left hand side as

$$\begin{aligned}
y^T(S(H+\sigma I)S)^\dagger y &= \sigma^{-1}y^T(\underbrace{\sigma^{-1}SHS}_{\equiv A} + SS)^\dagger y\\
&= \sigma^{-1}y^T\tilde{A}^\dagger y,
\end{aligned}$$

where we define $\tilde{A} = A + S^TS$. Note that the first fact also yields

$$SA^\dagger S = SA^\dagger = A^\dagger S = A^\dagger$$

Using the second fact, we then have

$$\begin{aligned}
\sigma^{-1}y^T\tilde{A}^\dagger y &= \sigma^{-1}y^T(A^\dagger - A^\dagger S(I + SA^\dagger S)^{-1}SA^\dagger)y\\
&= \sigma^{-1}y^T(A^\dagger - A^\dagger(I + A^\dagger)^{-1}A^\dagger)y\\
&= \sigma^{-1}y^T(A+I)^{-1}y\\
&= y^TS(SHS+\sigma I)^{-1}Sy
\end{aligned}$$

□

References

T. O. Lewis and T. G. Newman. Pseudoinverses of Positive Semidefinite Matrices. *SIAM Journal on Applied Mathematics*, 16(4):701–703, 1968. ISSN 0036-1399. URL `https://www.jstor.org/stable/2099121`.

George AF Seber. *A Matrix Handbook for Statisticians*, volume 15. John Wiley & Sons, 2008.
